# Health-related quality of life by household income in Chile: a concentration index decomposition analysis

**DOI:** 10.1186/s12939-022-01770-w

**Published:** 2022-12-13

**Authors:** Rodrigo Severino, Manuel Espinoza, Báltica Cabieses

**Affiliations:** 1 Unidad de Evaluación de Tecnologías en Salud, Centro de Investigación Clínica, Pontificia Universidad Católica de Chile, Facultad de Medicina, Santiago, Chile; 2 Department of Public Health, Faculty of Medicine, Pontificia Universidad Católica de Chile, Diagonal Paraguay 362, Piso 2, Santiago, Chile; 3 Instituto de Ciencias E Innovación en Medicina (ICIM), Facultad de Medicina, Universidad del Desarrollo, Santiago, Chile

**Keywords:** Concentration Index, Decomposition approach, Socioeconomic inequalities, Health-Related Quality of Life, EQ-5D, Chile

## Abstract

**Background:**

Health inequities have a profound impact on all dimensions of people’s lives, with invariably worse results among the most disadvantaged, transforming them into a more fragile and vulnerable population. These unfair inequalities also affect dimensions focused on subjectivity, such as health-related quality of life (HRQoL), which has been positioned, in recent decades, as an important outcome in health decision-making. The main objective of this study is to estimate socioeconomic inequality in HRQoL of Chilean by household income.

**Methods:**

Secondary analysis of the National Health Survey (ENS 2016–2017, Chile). This survey includes a nationally representative, stratified, and multistage household sample of people aged 15 and above. Socioeconomic inequality in HRQoL (EQ5D) is estimated by the concentration index (CI) ranked by household income. Decomposition analysis is conducted to examine potential explanatory sociodemographic factors.

**Results:**

The CI for household income inequality in HRQoL was -0.063. The lower the household income, the worse the HRQoL reported by in Chile. The decomposition analysis revealed that socioeconomic position contributes 75,7% to inequality in the quality of life, followed by educational level (21.8%), female gender (17.3%), and type of Health Insurance (15%), age (-19.7%) and residence (-10.8%). Less than 1% corresponds to the unexplained residual component.

**Conclusions:**

Our findings suggest the existence of a disproportionate concentration of worse HRQoL in the most disadvantaged socioeconomic groups in Chile. This inequality is largely, yet not completely, associated with household income. Other significant factors associated with this inequality are education, gender, and healthcare insurance. These results suggest the need of strengthening efforts to reducing socioeconomic gaps in health outcomes in Chile, as a means to achieve social justice and equity in health and healthcare.

## Background

Health inequalities have been defined as those measurable differences in health experience and outcomes between different population groups, according to socioeconomic level, geographic area, age, disability, gender, ethnic groups and others [1]. Reducing health inequalities has become a frequent topic of debate in the past decades. The United Nations (UN) considers them one of the sustainable development goals for 2030 [2] and has received a significant support in most countries in the world. These health inequalities are not a natural random phenomenon and have a profound impact on all dimensions of people’s lives, as they have strong influences in population health. As vastly documented, worse health results are found among those in socioeconomic disadvantage, as well as the concentration of their risk factors, revealing that the way societies are structured and stratified can have pervasive consequences in population health [3].

There is a large amount of literature that has explored the relationship between socioeconomic status and health outcomes, with a range of indicators to measure this association [4]. Of them all, income has consistently shown to have a strong influence in life opportunities [5]. Income inequality may have direct and indirect influences on population health. In terms of direct effects, it has been related to an unequal opportunity of obtaining good quality living conditions, avoiding environmental exposure, and buying quality food [6]. Indirect effects may be related to subjective sense of self-esteem and social value, individual health-risk behaviours that are socially taught, and others [7]. Income inequality has also been strongly linked to unequal opportunities of accessing health care and the quality of healthcare services received [8]. The scientific evidence is strong, societies with higher levels of inequality in income, education and other social determinants, have higher rates of infant mortality, abuse of illicit drugs, violence, obesity, imprisoners, adolescent pregnancy, less trust between people and less social cohesion [9].

Socioeconomic inequalities in population health have been measured for some health outcomes in Chile. In past decades, Chile has become a prosperous nation in the Latin American region [10]. However, despite experiencing an improvement in various indicators related to poverty and income, this country remains one of the most unequal countries in the region (e.g., Gini coefficient of 0,47 in year 2017) [11]. In terms of population health, the SALURBAL ecology study shows differences of up to 9 years in life expectancy in the city of Santiago for men, and 17.7 years for women, with worse results in the most vulnerable sectors [12]. Cabieses et al., using a representative national survey, found that there is a significant concentration of good-very good self-reported general health amongst those in the higher household income in the country [13]. Other studies have reported socioeconomic and income inequality in a number of health outcomes, such as in self-perceived oral health among adults in Chile, or income-related inequality in health and health care utilization in Chile [14]. The most disadvantaged older adults also have worse health outcomes, the prevalence and incidence of functional limitation follows a clear socioeconomic gradient in those older than 60 years [15]. The same occurs when analyzing other outcomes such as life expectancy or disability-free life expectancy, where it has been documented that people with a better socioeconomic position live longer and healthier lives compared to the more vulnerable population [16]. Even diseases as prevalent as Diabetes Mellitus II also follow a marked socioeconomic gradient when analyzed by quintiles of household income and educational level, where they also remain significantly associated with the presence of complications and attendance at health check-ups [17].

Health-related quality of life (HRQoL) has been positioned as an important outcome in healthcare decision-making, because it complements other more objective health indicators such as mortality or clinical effectiveness [18–20]. In aging populations with growing rates of chronic conditions, such as Chile and many other countries, HRQoL has raised as a useful measure of population health, and good predictor of the need for interventions and rising healthcare costs [21]. There are relevant studies in socioeconomic inequalities in HRQoL globally. A study in UK conducted by Davies et al. found that low SEP is a risk factor for hospital death as well as other indicators of potentially poor-quality end-of-life care [22]. Other studies have been published with similar findings in many countries, including some in the Latin American region [23–25].

Among many tools to measure HRQoL, EQ5D has become an instrument widely used. It has been defined as a generic questionnaire and has been used in several countries due to its simplicity, being included in some studies that have measured socioeconomic inequality in HRQoL in adult populations [26–29]. To the best of our knowledge, however, there is no study published analysing income inequality in HRQoL using the EQ5D instrument in the adult population in Chile. Therefore, our main objective is to measure the existing inequality HRQoL measured through EQ-5D by household income in adult population in Chile. For this, we estimated the concentration index and carried out a decomposition analysis that identifies a series of variables that were associated with the CI income inequality measure.

## Methods

### Data and sample

Secondary analysis of cross-sectional data from the National Health Survey of Chile (ENS 2016–2017); a nationally representative survey with a random, stratified, and multistage sample of 6233 people aged 15 and over [13]. Being a population survey with a complex sampling design, all estimates of the average design effect were made using weighting and the corresponding expansion factor. We assessed the social gradient of HRQoL in the adult population in Chile by socioeconomic status, particularly ranked household income, through the concentration index. In addition, we explored the potential effect of several covariates as determinants of these inequalities through a decomposition analysis of the concentration index. The ENS 2016–2017 survey is an anonymous database available per request by the Ministry of Health of Chile.

### Variables and measures

The dependent variable was HRQoL, measured by EQ-5D-3L and converted into cardinal values (also called health utilities) using the social value set validated for Chile in 2009 [30].

EQ-5D-3L assesses health status according to a descriptive system (questionnaire) and a Visual Analogue Scale (VAS). The dimensions evaluated by this questionnaire are Mobility, Self-Care, Usual Activities, Pain & Discomfort and Anxiety & Depression. Each dimension has 3 levels of severity: No problems, Moderate Problems, and Serious Problems, thus providing a final set of 243 health states [31]. The range of this variable considers negative values, which is a limitation for estimating the concentration index [32]. For this reason, the utilities obtained were transformed into disutilities, representing a decrease in utility (valued quality of life) due to a particular symptom, condition, or complication, as follows:

$${\varvec{D}}{\varvec{i}}{\varvec{s}}{\varvec{u}}{\varvec{t}}{\varvec{i}}{\varvec{l}}{\varvec{i}}{\varvec{t}}{\varvec{y}}\boldsymbol{ }=1-\left(\boldsymbol{ }{\varvec{U}}{\varvec{t}}{\varvec{i}}{\varvec{l}}{\varvec{i}}{\varvec{t}}{\varvec{y}}\right)$$

The independent variable was household income per capita. This was estimated from the ENS 2016–2017 dividing the household income by the number of members of the corresponding household. Control variables included in the decomposition analysis included educational level, household income, sex, age, area of residence (urban or rural), and the belonging to a particular health insurance system (public or private).

### Inequality measurement

We analysed income-related health inequalities examining gaps and gradients. First, we calculated the absolute and relative (20:20) gaps. Then, we depicted the inequality gradient using concentration curves. The Concentration Curve (CC) provides a means to evaluate the degree of inequality related to the socioeconomic position in the distribution of a health variable, plotting the cumulative percentage of the health variable on the y-axis (disutilities) against the accumulated percentage of the sample on the x-axis, ordered by their socioeconomic position, starting with the poorest and ending with the richest (as measured in this study by ranked household income per capita) [32]. While the concentration curve is important in depicting income-related inequality at each point in the income distribution for a health outcome of interest, it cannot be used to quantify the magnitude of such income-related inequality.

The magnitude of inequality in HRQoL is estimated using the Concentration Index (CI). The CI quantifies the degree of socioeconomic inequality of a health variable, displaying the health gradient in multiple subgroups with natural ordering or ranking. The CI expresses the extent to which an indicator of health is concentrated among the socially disadvantaged or the favoured [33]. The CI is defined as twice the area between the Concentration Curve and the equality line. The CI takes values between -1 and + 1, where a positive value indicates that a health variable is disproportionately concentrated among the most favoured people. In contrast, a negative value indicates the opposite (the variable is disproportionately concentrated in the most disadvantaged people), whereas a value of zero means that there is no inequality [34]. The sign of the concentration index indicates the direction of the relationship between the health variable and socioeconomic position, and its magnitude reflects both the strength of the relationship and the degree of variability in the health variable. The formula to calculate the concentration index is:

1 $${\varvec{C}}{\varvec{I}}=\frac{2}{{\varvec{\mu}}}{\varvec{c}}{\varvec{o}}{\varvec{v}}\boldsymbol{ }({{\varvec{\gamma}}}_{{\varvec{i}}}\boldsymbol{ },\boldsymbol{ }{{\varvec{R}}}_{{\varvec{i}}})$$

where γ_i_ denotes the health variable (EQ-5D results) of the i-th individual, μ it is average and R_i_ denotes the fractional rank of the ith individual concerning the socioeconomic position of their household. Given that we used disutilities instead of utilities, the CC in this analysis is expected to be drawn above the diagonal line and the CI is expected to be negative.

### Decomposition approach

According to Wagstaff et al. the CI can be decomposed into individual factors which contribute to -or are associated with- socioeconomic status related health inequality [35]. Each contribution corresponds to the product of the sensitivity of health concerning that factor and the degree of inequality related to income in that factor. In our study, to reveal the association of each explanatory variable to inequality in HRQoL, the approach of an additive linear regression model was used that links the results of the EQ-5D (*y*) to a set of *k* determinants:

2 $${{\varvec{\gamma}}}_{{\varvec{i}}\boldsymbol{ }}=\boldsymbol{ }\boldsymbol{\alpha }\boldsymbol{ }+\boldsymbol{ }\sum_{{\varvec{\kappa}}}{{\varvec{\beta}}}_{{\varvec{\kappa}}\boldsymbol{ }}{{\varvec{\chi}}}_{{\varvec{\kappa}}{\varvec{i}}}+{{\varvec{\varepsilon}}}_{{\varvec{i}}}$$

where *X*_*k*i_ is a set of *k* determining variables for the *i-th* individual, *β*_*k*_ is the coefficient or regressor and *ε* is the error term. Given the relationship between *γ*_*i*_ and *X*_*ki*_ in Eq. (2), the concentration index for *γ*, (CI), can be written as:

3 $${\varvec{C}}{\varvec{I}}=\boldsymbol{ }{\sum }_{{\varvec{\kappa}}}{({\varvec{\beta}}}_{{\varvec{\kappa}}\boldsymbol{ }}\overline{{{\varvec{\chi}} }_{{\varvec{\kappa}}}}/{\varvec{\mu}})\boldsymbol{ }{{\varvec{C}}}_{{\varvec{\kappa}}}+{\varvec{G}}{{\varvec{C}}}_{\begin{array}{c}\varepsilon \\ \end{array}}/{\varvec{\mu}}$$

where μ is the mean of γ, $\overline{{\chi }_{\kappa }}$ is the mean of ${\chi }_{\kappa }$, ${C}_{\kappa }$ is the concentration index for ${\chi }_{\kappa }$ (defined exactly as the concentration index for disutility), $\frac{{{\varvec{\beta}}}_{{\varvec{\kappa}}}\boldsymbol{ }\overline{{{\varvec{\chi}}}_{{\varvec{\kappa}}}}}{{\varvec{\mu}}}$ is the elasticity of disutility with explanatory variables and ${GC}_{\varepsilon }$ is the generalized concentration index for the residual component ε_i_.

## Results

### Sample description

The descriptive statistics are presented in Tables 1 and 2. The average age of the population under study was 49 years (95% CI 48,42—49,38) and women represent 62,9%. Only 11,15% of people lived in rural areas of Chile. Regarding the educational level, 24% had less than 8 years of formal education, and only 22% claimed to have more than 12 years of education. The household income per capita was described by quintiles. The difference between the averages of the most favoured and the least favoured quintile was USD 1,502 per capita. Further, 78.6% of the population were beneficiaries of the public health insurance. Regarding HRQoL measured by EQ-5D, the average utility for the study population is 0.786, while the average disutility (its complement) is 0,214.

**Table 1:** Summary statistics about HRQoL and its determinants in Chile, (ENS 2016–2017)

Variable (*n* = 6233 / Size of population 14.518.969)	Mean / %	Std. Err	[IC 95%]
**Age**	$$\overline{\mathrm{\rm X} }$$= 49		
15–24	18,8%	0,00897	[17,1-0,6]
25–44	37%	0,01227	[34,9-39,7]
45–64	31%	0,01056	[28,5-32,6]
65 +	13,2%	0,00686	[11,9-14,6]
**Gender**			
Female	50,8%	0,01129	[48,6-53,1]
Male	49,1%	0,01129	[46,8-51,3]
**Residence**			
Urban	88,84%	0,00690	[87,4-90,1]
Rural	11,15%	0,00690	[9,8-12,5]
**Socioeconomic Status (SES)** **Income Household Quintile ($USD)**^a^			
Q1	$$\overline{\mathrm{\rm X} }$$= 199,27		
Q2	$$\overline{\mathrm{\rm X} }$$= 339,34		
Q3	$$\overline{\mathrm{\rm X} }$$= 470,23		
Q4	$$\overline{\mathrm{\rm X} }$$= 710,91		
Q5	$$\overline{\mathrm{\rm X} }$$= 1.701,21		

^a^Per capita

**Table 2:** Summary statistics about HRQoL and its determinants in Chile, (ENS 2016–2017)

Variable (*n* = 6233 / Size of population 14.518.969)	Mean / %	Std. Err	[IC 95%]
**Educational Level**^a^			
≤ 8	24%	0,00945	[22,8-25]
8–12	54%	0,01401	[52,5-55]
12 +	22%	0,01434	[21,2-23,3]
**Type of Healthcare Provision**			
Public	78,5%		
FONASA—A	19,8%	0,00976	[18,21-21,6]
FONASA—B	21,7%	0,00963	[20-23,4]
FONASA—C	10,9%	0,00,699	[9,6-12,4]
FONASA—D	10,3%	0,00701	[9,1-11,6]]
Private (ISAPRE)	13,2%	0,00984	[11,5-14,9]
**HRQoL (EQ-5D)**			
Utility	$\overline{\mathrm{\rm X} }$= 0,766	0,00315	[0,759 – 0,771]
Disutility	$\overline{\mathrm{\rm X} }$= 0,234	0,00315	[0,228 – 0,240]

^a^Years of study

### Social gradient in HRQoL

**Fig. 1:**
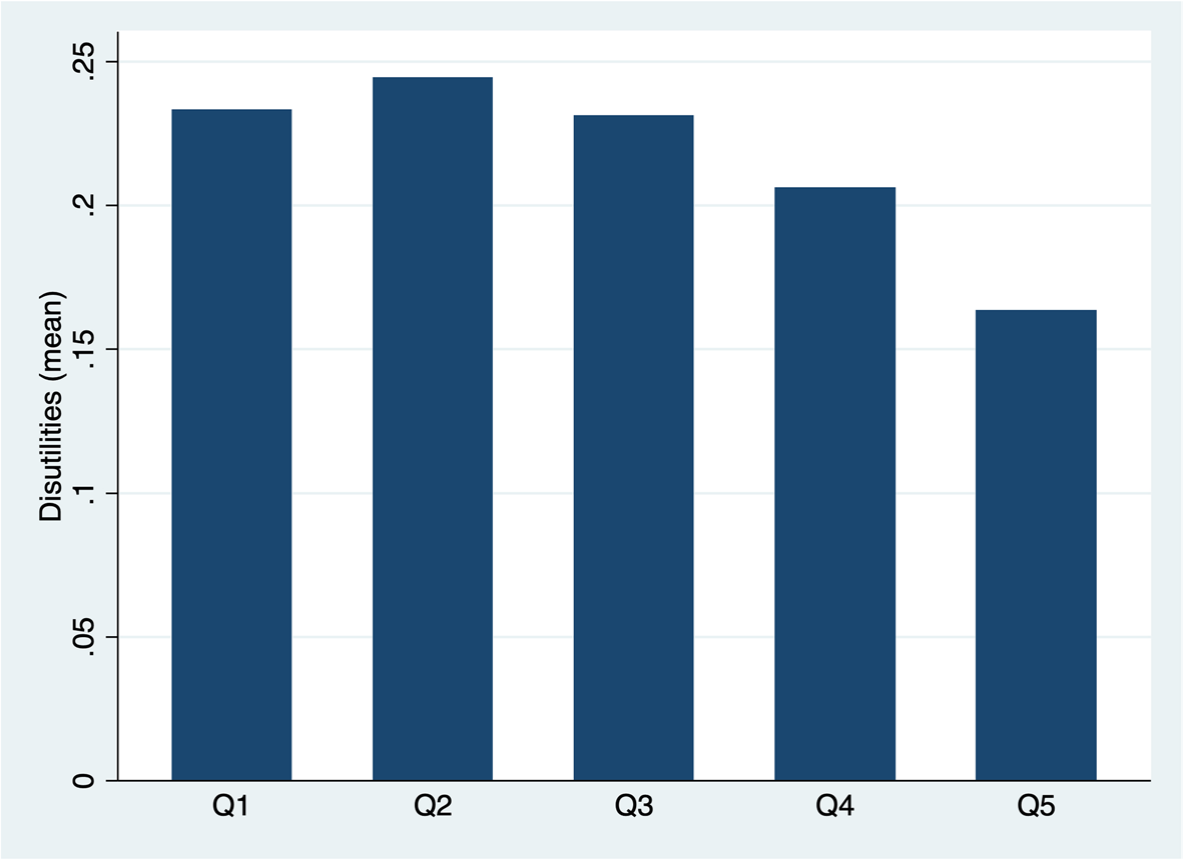
Distribution of disutilities by income per capita quintiles

Figure 1 shows the average of disutilities for each quintile of income per capita. It can be observed that the social gradient in health runs from the top to the bottom of the socioeconomic spectrum, concentrating the worst HRQoL in the most disadvantaged quintiles of the adult population in Chile.

### Absolute and relative inequality

Absolute and relative differences were calculated using the most extreme subgroups of socioeconomic position (Q1 and Q5) classified by age groups. In Table 3 we can see how each age interval concentrated the disutilities in the poorest quintile, representing 81% more for the 25–44 age range. This effect was somewhat diluted when the analysis was carried out considering all ages, where the concentration of disutilities in the most disadvantaged quintile represented an increase of 48%.

**Table 3:** Inequality ratio (20:20) and inequality gap (20–20) of mean disutility in Chile (ENS 2016—2017) by age groups

Age groups (years)	Poorest Quintile	Richest Quintile	Inequality Ratio 20:20	Inequality Gap 20–20
15-24	0,124	0,104	1,196	0,020
25-44	0,219	0,121	1,812	0,098
45-64	0,305	0,195	1,560	0,109
65 +	0,410	0,299	1,371	0,111
**All Ages**	**0,268**	**0,182**	**1,476**	**0,086**

### Concentration Curve (CC) and Concentration Index (CI)

**Fig. 2:**
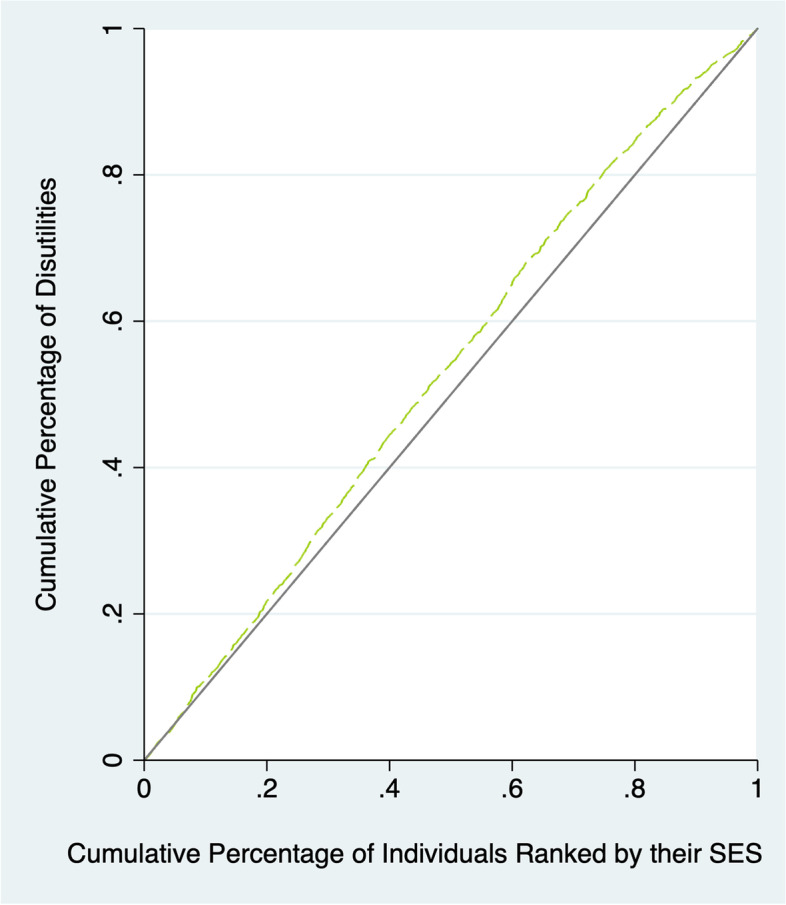
Health related quality of life concentration curve. SES: socioeconomic status

Figure 2 illustrates the HRQoL Concentration Curve. As mentioned above, the CC was above the equality curve, implying that the worst results were disproportionately concentrated in the most disadvantaged individuals. This finding was consistent with the results of the estimation of the Concentration Index shown in Table 4, which had a value of -0.063 (95% CI -0.091—-0.035), supporting the notion of disutilities were concentrated in the most disadvantaged quintiles of the adult population in Chile.

**Table 4:** Concentration Index (95% confidence interval, standard error and *P*-value) for HRQoL in Chile (ENS 2016–2017)

Method	Coef	Std. Error	95% Conf. interval	*P*-value
Convenient Regression	-0,063	0,014	[-0,091 – -0,035]	< 0,001
Convenient Covariance	-0,063	-	-	-

### Decomposition approach

When analysing factors associated with the CI and according to the detailed decomposition displayed in Tables 5 and 6, we found that income, as a proxy of socioeconomic position, contributes 76% to inequality in HRQoL, followed by educational level (22%), female gender (17%), and type of Health Insurance (15%). Of the total decomposition, less than 1% corresponded to the unexplained residual component, suggesting an adequate general fit of the decomposition model.

**Table 5:** Decomposition of Concentration Index for inequalities in HRQoL in Chile, ENS 2016–2017

Variable	Std. Err	Elasticity	CI	Absolute contribution	Percentage contribution
Age					
15 – 24^a^	-	-	-0,145	-	-
25—44	0,00,853	0,096	0,040	0,004	-6,02%
45—64	0,00,910	0,177	0,018	0,003	-5%
65 +	0,01,187	0,129	0,042	0,005	-8,69%
Gender					
Male^a^	-	-	-0,075	-	
Female	0,00,604	-0,144	0,077	-0,011	17,39%
Socioeconomic Status					
Q1	0,00,952	0,048	-0,795	-0,038	60%
Q2	0,00,915	0,063	-0,353	-0,022	35,31%
Q3	0,00,980	0,041	0,072	0,003	-4,64%
Q4	0,01,031	0,021	0,444	0,009	-14,97%
Q5^a^	-	-	0,813	-	-

^a^Denotes Reference Group

**Table 6:** Decomposition of concentration index for inequalities in HRQoL in Chile, ENS 2016–2017

Variable	Std. Err	Elasticity	CI	Absolute contribution	Percentage contribution
Educational Level					
≤ 8	0,01,138	0,046	-0,259	-0,012	18%
8–12	0,00,771	0,044	-0,071	-0,003	3,8%
13 + ^a^	-	-	0,339	-	-
Type of Healthcare Insurance					
Public	0,00,821	0,132	-0,072	-0,010	15,01%
Private^a^	-	-	0,314	-	-
Residence					
Urban	0,00,924	0,231	0,029	0,007	-10,82%
Rural^a^	-	-	-	-	-
Residual		-	-	0,001	0,53%
TOTAL				**-0,063**	**100%**

^a^Denotes Reference Group

Elasticities show the percentage of disutility change related to the average disutility, compared to a reference category. Thus, for example, the analysis of socioeconomic status indicates that, all quintiles between Q1 and Q4, compared to Q5, present an increase in disutility. The maximum change is observed between Q2 and Q5 (6.3%), which is consistent with the distribution of disutilities presented in Fig. 1.

## Discussion

This research tested the hypothesis that worse HRQoL was concentrated in the least advantaged socioeconomic groups in adult population in Chile, as measured by ranked household income per capita. Our findings support this idea, as we found a disproportionate concentration of worse HRQoL in the most disadvantaged socioeconomic groups in Chile, with a Concentration Index of -0.063. In the decomposition approach, we found that socioeconomic position was associated with socioeconomic inequality (household income per capita) (76%), but also other factors came to light in this analysis. Educational level (22%), female gender (17%), and type of Health Insurance (15%) were also associated with the CI of HRQoL in the adult population under study.

In addition, the decomposition shows that the contribution of different covariates to the total inequality is mainly explained by some sub-groups of the population. For example, for socioeconomic status, the poorest quintiles (Q1 and Q2) show a negative concentration index, whereas the remaining upper quintiles show positive concentration indices. This means that in Q1 and Q2 the disutility is concentrated in the relatively poorer people, but in Q3 to Q5 disutility is concentrated in those people relatively better-off. Indeed, we found that this concentration of disutility affecting more to the relatively richer people of every quantile, increases from Q3 to Q5. This interesting finding has been reported in previous studies [36].

This study is useful to current knowledge in the field of socioeconomic inequality in HRQoL and its findings are consistent with evidence conducted in other countries and regions. For example, a study conducted in Iran showed that a pro-rich distribution of better HRQoL among adults in the capitals of Kermanshah and Kurdistan Provinces [37]. A second study in China showed that SES was positively associated with HRQoL [38]. Similar findings have been reported in Chile for other health outcomes. For example, Cabieses et al. have already found a disproportionate distribution of good/very good self-reported general health in adult population in Chile using data from the CASEN survey, which supports the relevance of monitoring social inequalities in population health for different outcomes over time [13].

Our study has strengths and limitations. We value that this analysis was performed using an anonymous representative national-level dataset. It contained relevant variables for analysis, had very scarce missing values and included urban and rural areas of the territory. The statistical analysis used in this study was relevant to the estimation of social gradients in health and its related factors at population level. Hence, findings can inform national pending challenges in social inequalities in population health. We recognise as limitations of this study the cross-sectional nature of the dataset, which limits our interpretation of findings to associations but not causal inference. Some relevant variables were not included in the analysis like individual risk behaviours and health preferences, which could be expanded in the future using other datasets. Also, household income per capita could be complemented with other measures of socioeconomic status, such as those recommended by the OECD and the World Bank, and that were not available at the time of this study in this dataset because the data used (ENS 2016–2017) do not contain information on the individual characteristics of households (age of its members or their particular needs), which makes it impossible to generate models based on economies of scale such as corrected equivalent income.

In addition, our findings are relevant to health authorities, clinical practitioners and public health researchers in the country and the region. We found pervasive social inequalities in a complex and multidimensional health outcome, such as HRQoL. This is informative for health systems monitoring and vigilance of population health, as multiple costly health interventions are developed over time to reduce these and other social gaps in healthcare. This is the case of population-based screening programs, which are highly expensive, but they have the potential of impacting positively in term of reducing health inequalities in health outcomes when effective coverage is achieved [39–41]. In this context, because preventive services also show inequalities of access, monitoring their use is essential for an adequate interpretation [42]. Also, it is relevant as a baseline for future similar studies in the country and the region, as HRQoL has reached great attention and usefulness in subjective health in aging populations with high prevalence of chronic conditions. Findings are significant to decision making in healthcare, too. As indicated in this study, inequalities decrease the HRQoL of people throughout Chile, with potentially powerful economic, physical, and psychosocial effects. Therefore, the reduction of these social inequalities becomes an ethical imperative that must be transferred to robust public policies. Part of those inequalities relate to the healthcare system performance, but they are also determined by the organization of the system, which promote more structural inequalities in healthcare [43]. Nevertheless, because the determinants of inequalities (i.e., income, level of education, gender) cannot be tackled only by actions of the healthcare system, these policies need an intersectoral approach that points directly towards the most structural socioeconomic determinants of population health in Chile and the world.

## Conclusion

Evidence has demonstrated that income inequalities have harmful consequences in population health, including subjective dimensions of population health such as HRQoL. Our study supports this idea, as we found a disproportionate concentration of worse HRQoL in the most disadvantaged socioeconomic groups in Chile, with a Concentration Index of -0.063. Furthermore, in the decomposition approach, we found that socioeconomic position was widely associated with inequality (76%), but also other factors came to light in this analysis. Educational level (22%), female gender (17%), and type of Health Insurance (15%) were also associated with the CI of HRQoL in the population under study. These results provide a measure of health inequality to monitor the progress of social justice in health policy making in Chile, and also offers a relevant benchmark for international comparative analysis, especially with other middle and low-income countries. 

## Data Availability

All data generated or analysed during this study are included in this published article.

## Abbreviations

HRQoL: Health-Related Quality of Life
EQ-5D: Euro-Quality of Life – Five Dimensions
ENS: Encuesta Nacional de Salud (National Health Survey)
UN: United Nations
CC: Concentration Curve
CI: Concentration Index
Q1-5: Quintile 1- Quintile 5
OECD: Organisation for Economic Cooperation and Development
